# Natural limestone discolouration triggered by microbial activity—a contribution

**DOI:** 10.3934/microbiol.2018.4.594

**Published:** 2018-08-10

**Authors:** Luís Dias, Tânia Rosado, Ana Coelho, Pedro Barrulas, Luís Lopes, Patrícia Moita, António Candeias, José Mirão, Ana Teresa Caldeira

**Affiliations:** 1 HERCULES Laboratory, University of Évora, Largo Marquês de Marialva 8, 7000-089 Évora, Portugal; 2 Chemistry Department, Sciences and Technology School, University of Évora, Rua Romão Ramalho 59, 7000-671 Évora, Portugal; 3 Institute of Earth Sciences, University of Évora, Rua Romão Ramalho 59, 7000-671 Évora, Portugal; 4 Geosciences Department, Sciences and Technology School, University of Évora, Rua Romão Ramalho 59, 7000-671 Évora, Portugal

**Keywords:** building stone, limestone, alteration, colour, microbial contamination

## Abstract

Colour is a major argument that drives the decision of an architect in a specific architecture project and one of the most important characteristics and perceptible aspects of natural building stones. “Blue” limestones are building rocks, with different geological ages, typically used in several countries, and are known for their vulnerability to alteration, which causes colour change and the occurrence of unaesthetic patterns. Owing to this vulnerability, the conservation-restoration works in monuments, or new buildings constructed with “blue” limestone is extremely costly. Considering that the main limitation of this lithological variation is the chromatic change, a multidisciplinary approach was envisaged in this study to allow a closer insight into the chemical and mineralogical alterations and the microbial communities. Results obtained suggest that the inorganic alteration in the “blue” limestone may create favourable conditions for microbial growth and could lead to an increment in deterioration process.

## 1. Introduction

Building stone is probably the most visible material in built cultural heritage, its characteristics remain in the memory of travellers long after their visit. The conservation of such buildings is a major problem requiring permanent monitoring and expensive maintenance and restoration works. Colour is one of the most important characteristics and visible aspects of natural building stones. Since discolouration is a critical issue of this material, the colour and the discolouration of carbonate rocks is also a major scientific problem with extremely important economic consequences in historic monuments and new buildings.

The inorganic processes of rock weathering are well-known, but in the ornamental stones framework, where aesthetic details are essential, the influence of human activities and commercial surface finishing [1], such as polished, flamed, brushed and sandblasted finishing, among others, may lead to different decay patterns.

Furthermore, the external colonisation of building surfaces by microorganisms causes unacceptable unaesthetic alterations on the stone surfaces, namely staining appearance promoted by biogenic pigments [2],[3]. Moreover, the inorganic weathering can create conditions that will contribute to the growth of biological colonisation, thereby increasing the rate of alteration. Hence, it is of utmost importance to reiterate the mechanisms of inorganic-biological alteration.

Microbial communities have very distinct and diverse patterns that can be linked to different environmental conditions. Therefore, microbial colonisation and deterioration of stones are intimately connected to the changes in the environment [4]–[6]. The most significant cited parameters affecting microbial colonisation are physical factors, mainly humidity, temperature, light intensity, and the composition of air pollutants, as well as the chemical nature, mechanical strength, solubility and porosity of the substratum [7],[8]. In general, microbial colonisation is initiated by a wide variety of phototrophic microorganisms, mostly cyanobacteria and algae [9]–[11]. The colonisation of stone monuments by light-dependent phototrophic types, including cyanobacteria, algae and lichens, allows organic matter in the form of dead cells and trapped debris to be accumulated, enabling heterotrophic or chemoorganotrophic fungi and bacteria to grow on the surface of the stone [12].

**Figure 1.**
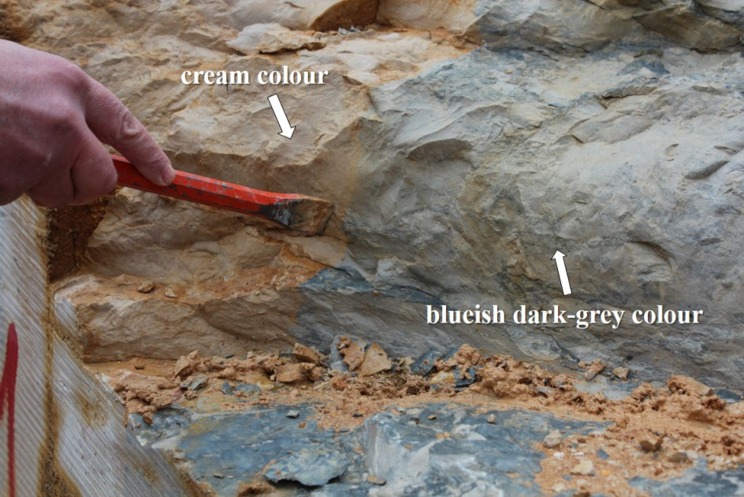
“Blue” limestone with blueish dark-grey and cream colour in the same outcrop.

“Blue” limestone is a building rock, with different geological ages, typically used in several countries, like Portugal, Belgium, China, Ireland and Vietnam. The name is derived from the blueish dark-grey colour associated with the presence of organic matter. This good quality natural stone coexists with a cream-coloured limestone, sometimes in the same outcrop (Figure 1), and it remains unclear which factors lead to the occurrence of each colour.

**Figure 2.**
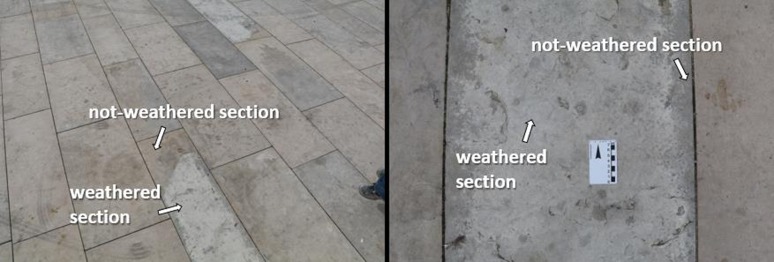
Outdoor pavement in “blue” limestone with visible alteration.

“Blue” limestone is also susceptible to unknown weathering processes, that cause colour change and promote the occurrence of unaesthetic patterns (Figure 2). The main objective of this study is to identify how the changes in chemical and mineralogical composition in the “blue” limestone can be correlated with the microbial colonisation state. Considering the same micro-climatic conditions, sections of weathered rock are compared with not-weathered sections to determine both chemical and mineralogical changes and evaluate the biocolonisation susceptibility.

## 2. Materials and methods

### 2.1. Sampling process

Sampling was performed in a “blue” limestone outcrop for stone characterisation; fragments of blueish dark-grey and cream coloured limestone were recovered. Additionally, building stone displaying chromatic alteration were sampled. Micro-fragments from outdoor building stones were also collected for microbiological assessment in weathered and not-weathered sections. Due to rock heterogeneity and in order to avoid an excessive number of variables, sampling was performed in closely related altered and non-altered zones, ensuring representativeness.

The collection for microbial assessments was done under semi-aseptic conditions with sterile scalpels and microtubes and conserved at 4 °C until utilisation.

### 2.2. Material characterisation

#### 2.2.1. X-ray diffraction (XRD)

The mineralogical characterisation was performed by X-ray diffraction with a Bruker D8 Discover microdiffractometer system equipped with a linear detector Bruker Lynxeye, a Goebel mirror, a laser-video sample alignment system and a motorized XYZ stage. Diffraction data were recorded using CuKα radiation, tube running conditions as 40 kV and 40 mA. The XRD patterns were measured in the range 3° to 75° 2θ with a step size of 0.05° and a recording time of 1 s per step. The identification of the crystalline phases was performed with the PDF-ICDD Powder Diffraction Database (International Centre for Diffraction Data) and the Bruker EVA software (version 3.0).

The XRD experiments were carried out on the exposed surface of the stone, using a 1 millimetre X-ray collimator. The mineralogical characterisation of the samples of the two coexisting colours of the “blue” limestone outcrop was performed in macro mode, with previous rock powdering. On the other hand, the characterisation of the alteration products was performed in micro mode, on the surface of the outdoor building stones, in closely related areas with and without alteration patterns.

#### 2.2.2. Portable X-ray fluorescence spectrometry (XRF)

To perform chemical and phase composition, X-ray fluorescence spectrometry analyses were done with a portable Bruker Tracer III-SD at low vacuum conditions, using an XFlash^®^ SDD detector with 145 eV of resolution. The X-ray generator was operated at 40 kV and 30 µA current, with an acquisition time of 120 s. The data analysis software ARTAX 7.4.0 was used in order to identify the different X-ray emission peaks.

#### 2.2.3. Variable pressure scanning electron microscopy with energy dispersive spectroscopy (VP-SEM-EDS)

VP-SEM-EDS was performed with a HITACHI S3700N interfaced with a QUANTAX EDS microanalysis system, equipped with a BRUKER XFlash^®^ Silicon Drift Energy Dispersive Detector with 129 eV spectral resolution at FWHM/Mn Kα. The EDS data was processed in the Espirit1.9 software using the standardless tools. The backscattering mode was employed to detect slight changes in the composition of the surface materials. When the surface was chemically heterogeneous, EDS compositional maps was produced and point analyses were obtained. The operating conditions in backscattered mode and EDS analyses were performed as follows: 40 Pa chamber atmosphere, 20 kV accelerating voltage and 10–12 mm working distance.

### 2.3. Microbiological assessment

#### 2.3.1. Assessment of biological contamination on outdoor building stones

Stone fragments collected were coated with a gold/palladium layer in an SCD030 Balzers Union sputter-coater, and the surface of the rock was carefully examined in Scanning Electron Microscopy described in 2.3.2., with an accelerating voltage of 10 keV in secondary electrons mode, to assess the microbial communities presence.

#### 2.3.2. Cell viability index

Cell viability index (CVI) of the biocontaminants present on the stone materials was assessed, according to the procedure previously described by Mosmann [13], by 3-(4,5-dimethylthiazol-2-yl)-2,5-diphenyltetrazolium bromide (MTT) assay.

A 90 µL of each sample extracted suspension from outdoor building stones with and without chromatic alteration were incubated with 300 µL of MTT stock solution (0.5 mg/mL) for 4 h, at 37 °C in the dark. After this, 350 µL of DMSO/ethanol (1:1) was added to dissolve the formazan crystals formed. The final suspension was centrifuged at 10,000 rpm for 15 min and the absorbance of the supernatant was determined by spectrophotometry at 570 nm. This method was performed in seven different areas with and without chromatic alteration, and each assay was performed in triplicate.

#### 2.3.3. Characterisation of the microbial population

Metagenomic DNA was extracted from outdoor building stone samples using QIAmp DNA Stool Mini Kit (Qiagen, Limburg, Netherlands), with slight modifications from the manufacturer's instructions.

Bacterial and fungal communities were characterised by high-throughoutput sequencing (HTS) for the 16S rRNA V3–V4 region and Internal Transcribed Spacer 2, respectively, using Illumina Sequencing platform.

DNA was amplified for the hypervariable regions with specific primers and further reamplified in a limited-cycle PCR reaction to add sequencing adaptor and dual indexes. First, PCR reactions were performed for each sample using 2× KAPA HiFi HotStart Ready Mix. In a total volume of 25 µL, 12.5 ng of template DNA and 0.2 µM of each PCR primer.

For bacteria the following primers were used: forward primer Bakt_341F 5′-CCTACGGGNGGCWGCAG-3′ and reverse primer Bakt_805R 5′-GACTACHVGGGTATCTAATC C-3′ [14],[15]. For fungi, a pool of forward primers was used: ITS3NGS1_F 5′-CATCGATGAAGAACGCAG-3′, ITS3NGS2_F 5′-CAACGATGAAGAACGCAG-3′, ITS3NGS3_F 5′-CACCGATGAAGAACGCAG-3′, ITS3NGS4_F 5′-CATCGATGAAGAACGTAG-3′, ITS3NGS5_F 5′-CATCGATGAAGAACGTGG-3′, and ITS3NGS10_F 5′-CATCGATGAAGAACGCTG-3′ with the reverse primer ITS3NGS001_R 5′-TCCTSCGCTTATTGATATGC-3′ [16]. The PCR conditions involved 3 min of denaturation at 95 °C, followed by 25 cycles of 98 °C for 20 s, 55 °C for 30 s and 72 °C for 30 s and a final extension at 72 °C for 5 min. Negative controls were included for all amplification reactions. Electrophoresis of the PCR products was undertaken on a 1% (w/v) agarose gel and the ∼490 bp V3–V4 and ∼390 bp ITS2 amplified fragments were purified using AMPure XP beads (Agencourt, Beckman Coulter, USA) according to manufacturer's instructions. Second PCR reactions added indexes and sequencing adaptors to both ends of the amplified target region by the use of 2× KAPA HotStart Ready Mix, 5 µL of each index (i7 and i5) (Nextera XT Index Kit, Illumina, San Diego, CA) and 5 µL of the first PCR product in a total volume of 50 µL. The PCR conditions involved a 3 min denaturation at 95 °C, followed by 8 cycles of 95 °C for 30 s, 55 °C for 30 s and 72 °C for 30 s and a final extension at 72 °C for 5 min. Electrophoresis of the PCR products was undertaken on a 1% (w/v) agarose gel and the amplified fragments were purified using AMPure XP beads (Agencourt, Beckman Coulter, USA) according to manufacturer's instructions.

The amplicons were quantified by fluorimetry with PicoGreen dsDNA quantitation kit (Invitrogen, Life Technologies, Carlsbad, California, USA), pooled at equimolar concentrations and paired-end sequenced with the V3 chemistry in the MiSeq® according to manufacturer's instructions (Illumina, San Diego, CA, USA) at Genoinseq (Cantanhede, Portugal). They were multiplexed automatically by the Miseq® sequencer using the CASAVA package (Illumina, San Diego, CA, USA) and quality-filtered with PRINSEQ software [17] using the following parameters: (1) bases with average quality lower than Q25 in a window of 5 bases were trimmed, and (2) reads with less than 220 bases were discarded for V3–V4 samples and less than 100 bases for ITS2 samples.

The forward and reverse reads were then merged by overlapping paired-end reads using the AdapterRemoval v2.1.5 [18] software with default parameters. The QIIME package v1.8.0 [19] was used for Operational Taxonomic Units (OTU) generation, taxonomic identification and sample diversity and richness indexes calculation.

Sample IDs were assigned to the merged reads and converted to fasta format (split_libraries_fastq.py, QIIME). Chimeric merged reads were detected and removed using UCHIME [20] against the Greengenes v13.8 database [21] for V3–V4 samples and UNITE/QIIME ITS v12.11 database [22] for IST2 samples (script identify_chimeric_seqs.py, QIIME).

OTUs were selected at 97% similarity threshold using the open reference strategy. First, merged reads were pre-filtered by removing sequences with a similarity lower than 60% against Greengenes v13.8 database for V3–V4 samples and UNITE/QIIME ITS v12.11 database for ITS2 samples. The remaining merged reads were then clustered at 97% similarity against the same databases listed above. Merged reads that did not cluster in the previous step were again clustered into OTU at 97% similarity. OTUs with less than two reads were removed from the OTU table. A representative sequence of each OTU was then selected for taxonomy assignment (pick_rep_set.py, assign_taxonomy.py; QIIME).

## 3. Results and discussion

In order to try to understand the relation between biocolonisation and weathering of this type of limestone—key-factors that strongly affect the aspect and integrity of the stone leading to costly conservation work, a multidisciplinary approach was applied. Complementary analytical methodologies for the characterisation of material and alteration products, combined with colonisation state assessment, contributed with relevant information to comprehend the association between chemical and mineralogical alteration and biological colonisation in the rock surface, under the same micro-climatic conditions.

### 3.1. Chemical and mineralogical composition and superficial texture

**Figure 3.**
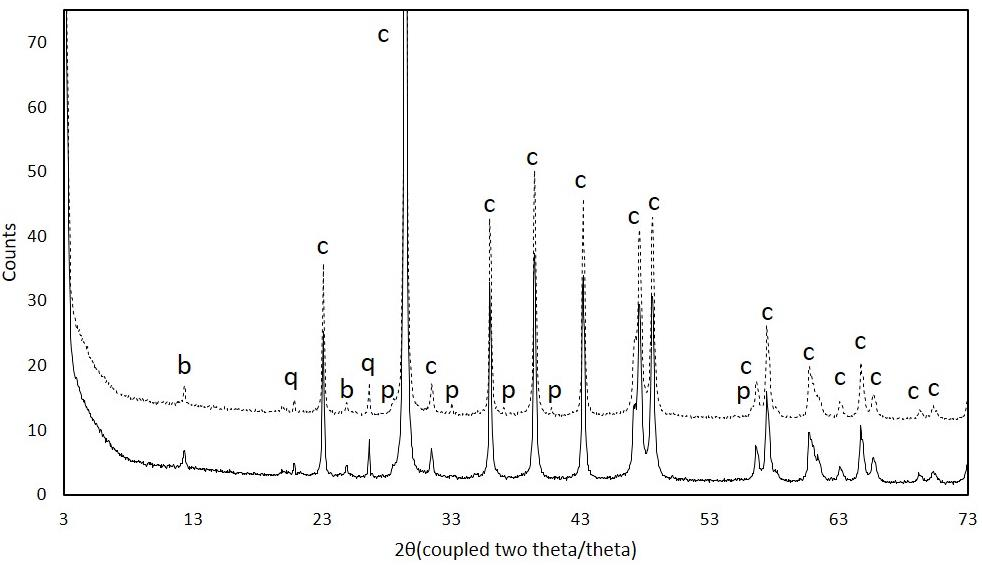
Diffractograms showing the presence of different mineralogical phases in cream coloured zones (^____^) and blue coloured zones (----) of the samples recovered from the “blue” limestone outcrop. Abbreviations: b—birnessite; q—quartz; c—calcite; p—pyrite.

Comparative data between blue and cream coloured zones of the samples recovered from the “blue” limestone outcrop show that calcite is, as expected, the most abundant mineralogical phase, quartz is always present, and pyrite (FeS_2_) and birnessite (Na_0.55_Mn_2_O_4_·1.5H_2_O) are present in the darker fraction of the stone (Figure 3). X-ray fluorescence data showed a slight increment in sulphur in the darker fraction, corroborating the presence of pyrite.

**Figure 4.**
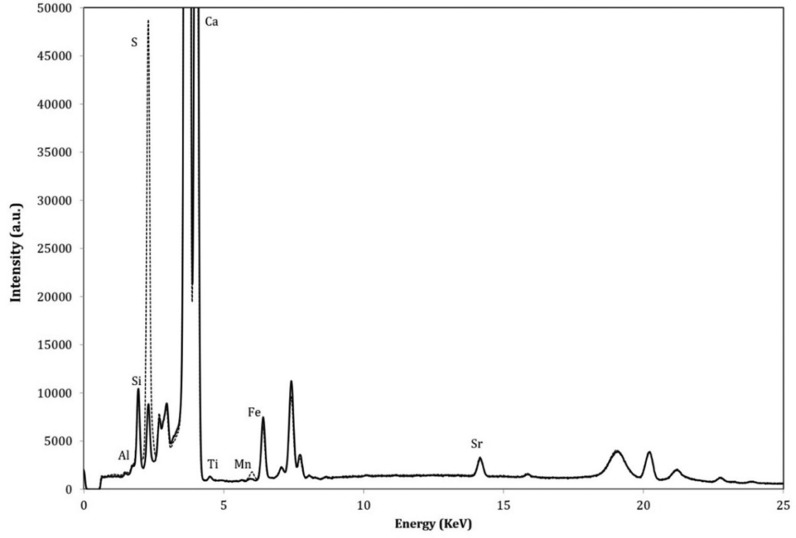
Compositional spectra by X-ray fluorescence from the sample with chromatic alteration in outdoor building stone, in altered (----) and non-altered areas (^_____^).

Samples from outdoor building stone were firstly analysed by portable X-ray fluorescence, in chromatic altered and non-altered areas. The elements found, such as Ca, Si, Al, K, Sr, Fe and Mn are common in limestones and seems to be present in similar quantities both with or without chromatic alteration areas. Nevertheless, in the spectrum from chromatically altered area stands out a clear enrichment in sulphur (Figure 4).

**Figure 5.**
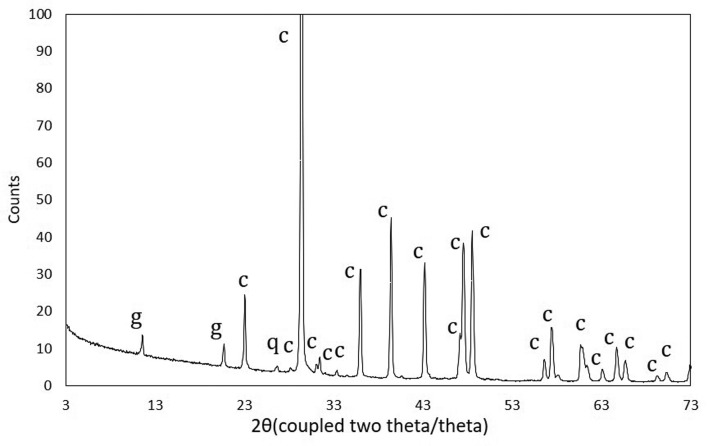
Micro-X-ray diffractogram obtained on the altered area from building stone. Abbreviations: g—gypsum; c—calcite; q—quartz.

**Figure 6.**
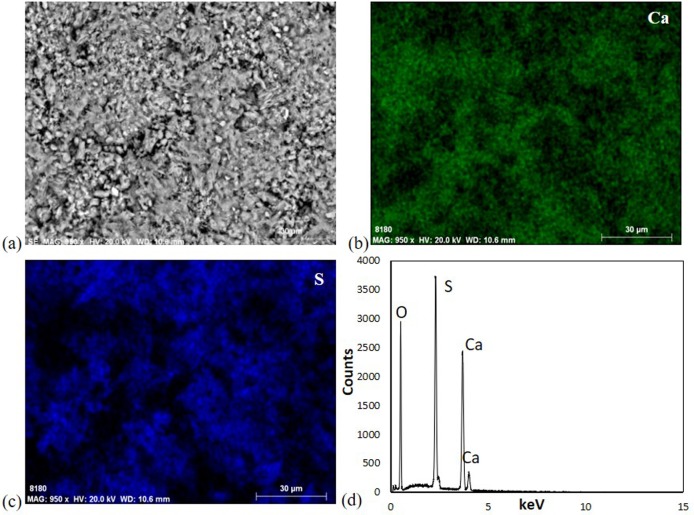
(a) Microstructure, element mapping of (b) calcium and (c) sulphur, and point analysis in limestone weathered section, performed by VP-SEM-EDS.

Micro-X-ray diffraction performed on chromatically altered areas revealed an enrichment in gypsum (CaSO_4_·2H_2_O), as demonstrated in Figure 5. The presence of gypsum in altered surface zones was confirmed by VP-SEM-EDS, with the coexistence of calcium and sulphur in the same regions, as it is shown in bi-dimensional elemental mapping and point analysis (Figure 6).

**Figure 7.**
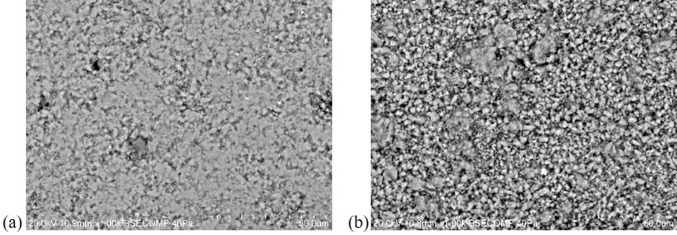
Limestone microstructure in (a) non-altered surface and (b) altered surface, by VP-SEM-EDS.

Additionally, this technique also evidenced differences in the surfaces' microstructure, where the surface with chromatic alterations seems less compact (Figure 7), possibly due to the polishing loss. This may lead to a favourable condition to microbial proliferation on the stone surface [23].

### 3.2. Microbial evaluation

**Figure 8.**
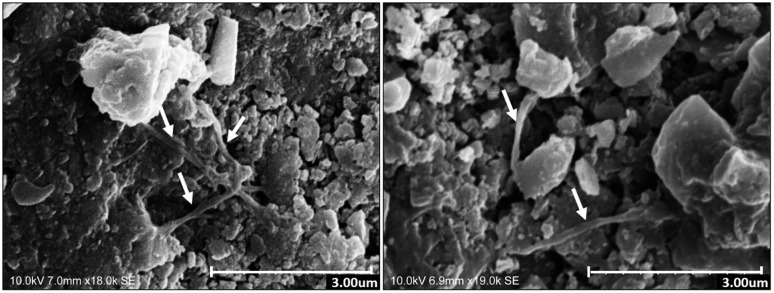
SEM micrographs in weathered sections. Arrows signalize hyphae of filamentous fungi proliferating around calcite crystals.

SEM analysis allowed a further insight into the presence of microbial communities thriving on the studied outdoor building stones with chromatic alteration and their capacity to proliferate within the stone surface (Figure 8). The micrographs show the presence of filamentous fungi and spores on these surfaces. Using this technique, no microorganisms were found in non-altered surfaces.

**Figure 9.**
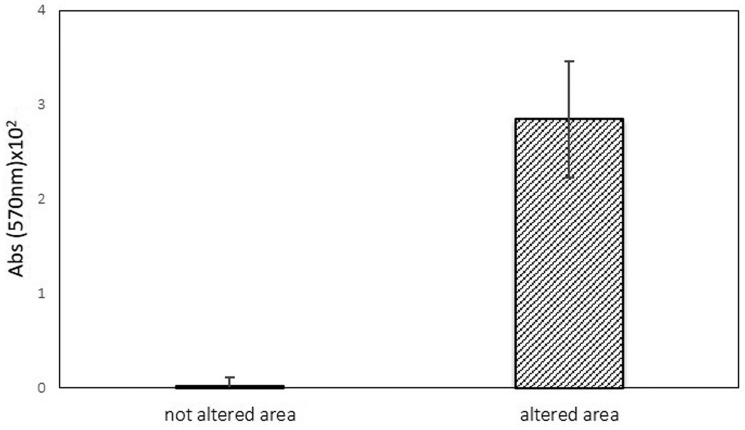
Cell viability of the microbial population present in altered areas and non-altered areas, of building stone. Error bar corresponds to ±standard deviation (n = 21).

Regarding the cell viability index, the method used showed that samples from chromatically altered areas seem to have higher CVI levels, than that of the non-altered areas (Figure 9). High CVI degree potentiates metabolic activity. This method was previously optimised to biodeterioration on cultural heritage materials and has been found to be simple, fast, and very sensitive [24], giving an overview of the presence of microorganisms and also a preliminary screening of their metabolic activity.

**Figure 10.**
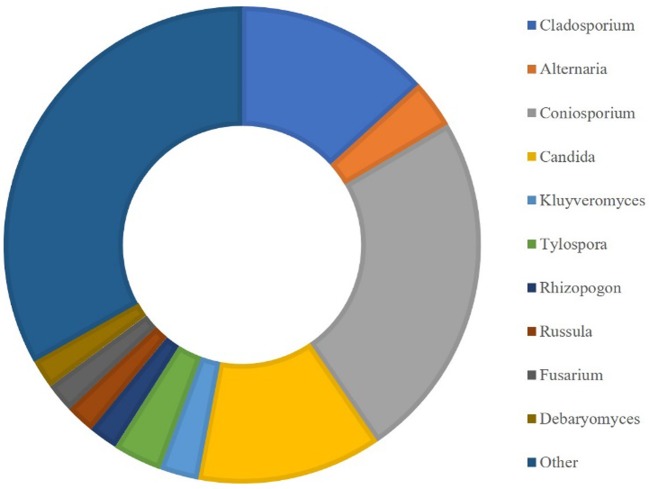
Characterisation of the eukaryotic population at genera level present in limestone altered areas.

**Figure 11.**
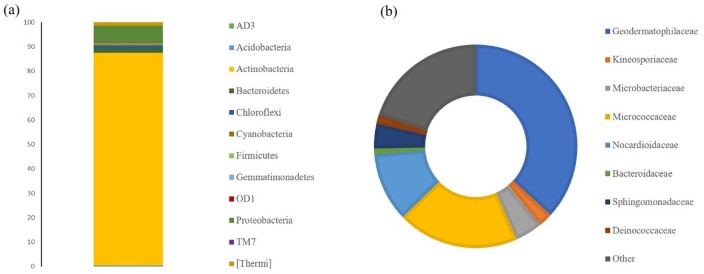
Characterisation of the prokaryotic population present in limestone altered areas at (a) phylum and (b) family levels.

HTS allowed the detailed characterisation of the microbial population present on limestone altered areas (Figures 10 and 11). For eukaryotic population around 55,000 OTU were generated and the predominant genera identified (Figure 10) were *Coniosporium* (3.6%) and *Cladosporium* (2.0%), both belonging to Ascomycota phylum commonly associated with rock substrate [25]. On the other hand, the prokaryotic population generated around 70,800 OTU, whose dominant phyla (Figure 11a) were Actinobacteria (87.2%) and Proteobacteria (7.1%), while the prevalent families (Figure 11b) were Geodermatophiliceae (36.8%), Micrococcaceae (19.4%) and Nocardioidaceae (10.9%). Actinobacteria was previously associated with biofilms formation in siliceous stone as major colonisers [26]. Other less representative phyla, like Firmicutes, were found. Some bacterial strains of the Firmicutes phylum have been found to reproduce biodeterioration in laboratory studies [27]. The most abundant genus is *Modestobacter* (25.3%) and has been previously correlated with deterioration of cultural heritage limestones [28], and in other types of stone and has been shown to be capable of surviving on extreme environmental conditions [29]. Other less representative genera were also identified, such as *Geodermatophilus*, *Agrococcus*, *Arthrobacter* and *Deinococcus*. According to these results, it seems that the main biocolonisers of these materials are bacterial communities.

These two different approaches, HTS and CVI, encompass and give useful information about the presence of microorganisms and their metabolic activity, allowing the characterisation of the microbial fauna [30], and provided an important and detailed description of the communities able to develop on the “blue” limestone.

The results indicate that, perhaps, these changes on the rock mineralogy by weathering can provide favourable conditions to microbial proliferation on the damaged surface. Moreover, microbial proliferation and activity may increase structural damages on the surface of the rock.

## 4. Conclusions

The discolouration of “blue” limestone is a major problem that affects this highly priced natural product vastly used as building stone in major contemporary architecture projects and in the past, in important monuments. This study focused on the weathering of “blue” limestone, particularly on the discolouration process and the possibility of the further susceptibility of weathered areas to promote biocolonisation in its surface.

Data obtained revealed the presence of great amounts of gypsum on the altered surfaces, when compared with surfaces without alteration in the same limestone. The formation of this sulphate seems to change the surface microstructure, leading to polishing loss and an increase in roughness. Regarding the microbial contamination, it was demonstrated that the surfaces with visible alteration were predominantly more contaminated, with higher CVI.

According to HTS results, it was demonstrated that the major bacteria present belong to the phylum Actinobacteria and may have an important role on colonisation within the material.

Results suggest that the alteration that occurs in “blue” limestone may create favourable conditions to microbial growth, due to an increase in surface roughness and further enhancement of microorganisms capacity to anchor and penetrate within the damaged surface. Therefore, the inorganic initial surface alteration can promote the biocolonisation that will increase the deterioration rate, creating a cause-effect cycle. This study constitutes an important contribution to understand building limestone deterioration and drive us to continue investigating the alteration mechanism, to predict and prevent this chromatic alteration.
